# Supplementary material for: Association of School Instructional Mode with Community COVID-19 Incidence during August–December 2020 in Cuyahoga County, Ohio
Source: Int J Environ Res Public Health. 2024 Apr 29;21(5):569. doi: 10.3390/ijerph21050569 (PMC11121418; doi:10.3390/ijerph21050569)
Supplement: Supplementary file 1 [file ijerph-21-00569-s001.zip › ijerph-2931440-supplementary.pdf]

Supplement—Terebuh et al

Supplementary Materials and Methods
Table S1
References

Supplementary Materials and Methods

The Social Vulnerability Index (SVI) has been developed as a public health planning and preparedness tool for the distribution of resources. "Social vulnerability refers to the potential negative effects on communities caused by external stresses on human health." Disease outbreaks were one of the scenarios for which the SVI was created. The composite SVI reflects a census tract's percentile rank ranging from 0 to1, with a higher value corresponding to greater vulnerability. The composite SVI is constructed by combining the rankings of the 15 individual U.S. census variables listed below.

- Socioeconomic Status
  - Below Poverty
  - Unemployed
  - Income
  - No High School Diploma
- Household Characteristics
  - Aged 65 & Older
  - Aged 17 & Younger
  - Civilian with a Disability
  - Single-Parent Households
  - English Language Proficiency
- Racial & Ethnic Minority Status
  - Hispanic or Latino (of any race); Black and African American, Not Hispanic or Latino; American Indian and Alaska Native, Not Hispanic or Latino; Asian, Not Hispanic or Latino; Native Hawaiian and Other Pacific Islander, Not Hispanic or Latino; Two or More Races, Not Hispanic or Latino; Other Races, Not Hispanic or Latino
- Housing Type & Transportation
  - Multi-Unit Structures
  - Mobile Homes
  - Crowding
  - No Vehicle
  - Group Quarters

Table S1. Zip code total population and social vulnerability index.

Zip Code[1]	Total Population	Social Vulnerability Index[2]	Instructional Mode[3]
44017	18903	0.39	NRI
44040	2903	0.01	NRI
44070	31847	0.33	NRI
44102	45514	0.85	RI
44103	16519	0.90	RI
44104	19640	0.94	RI
44105	37211	0.80	RI
44107	50666	0.34	RI
44108	21939	0.82	RI
44109	40246	0.95	RI
44110	19116	0.87	RI
44111	42821	0.71	RI
44112	21909	0.85	RI
44113	20358	0.61	RI
44114	6567	0.87	RI
44115	8284	0.76	RI
44116	20338	0.24	NRI
44117	9611	0.67	NRI
44118	38833	0.41	RI
44123	16515	0.56	NRI
44126	16372	0.22	NRI
44127	4375	0.91	RI
44129	28276	0.36	NRI
44132	14805	0.69	NRI
44133	30225	0.27	RI
44134	37533	0.42	NRI
44135	27640	0.71	RI
44136	25776	0.28	NRI
44137	22657	0.64	RI
44138	22378	0.19	NRI
44139	24212	0.34	NRI
44140	15391	0.06	NRI
44141	13830	0.13	NRI
44142	18674	0.55	NRI
44145	32378	0.31	NRI
44146	29223	0.55	RI
44149	18976	0.10	NRI
Range	(2903 – 45514)	(0.0177 – 0.9523)	RI mean = 0.7294 (0.2726 – 0.9523) NRI mean = 0.3304 (0.0177 – 0.6969)

[1]Zip codes straddling more than one school district (>10%) were excluded from the analysis.
[2]Social Vulnerability Index estimate was population weighted by U.S. Census Tract within each zip code. U.S. Census Tract populations that straddled zip codes were assigned to the majority zip code unless the population majority was evenly divided (within 60:40). Those census populations were split between the two zip codes.
[3]Zip codes in school districts that remained in remote mode (RI) during the entire study period; non-remote instructional mode (NR) included in this category if they employed any non-remote schooling during the study period.
RI = remote instruction; NRI = non-remote instruction

Supplementary References

1. CDC/ATSDR Social Vulnerability Index. https://www.atsdr.cdc.gov/placeandhealth/svi/index.html
2. CDC/ATSDR SVI Data and Documentation Download. https://www.atsdr.cdc.gov/placeandhealth/svi/data_documentation_download.html
3. Flanagan B, Gregory E, Hallisey E, et al. A social vulnerability index for disaster management. Journal of Homeland Security and Emergency Management. 2011;8(1). https://www.atsdr.cdc.gov/placeandhealth/svi/img/pdf/Flanagan_2011_SVIforDisasterManagement-508.pdf
